# Population-specific association between *ABCG2* variants and tophaceous disease in people with gout

**DOI:** 10.1186/s13075-017-1254-8

**Published:** 2017-03-07

**Authors:** Wendy He, Amanda Phipps-Green, Lisa K. Stamp, Tony R. Merriman, Nicola Dalbeth

**Affiliations:** 1 Department of Medicine, Faculty of Medical and Health Sciences, University of Auckland, 85 Park Rd, Grafton, Auckland New Zealand; 2 Department of Biochemistry, University of Otago, Dunedin, New Zealand; 3 Department of Medicine, University of Otago, Christchurch, New Zealand

**Keywords:** Gout, Tophus genetics, *ABCG2*

## Abstract

**Background:**

Tophi contribute to musculoskeletal disability, joint damage and poor health-related quality of life in people with gout. The aim of this study was to examine the role of *SLC2A9* and *ABCG2* variants in tophaceous disease in people with gout.

**Methods:**

Participants (n = 1778) with gout fulfilling the 1977 American Rheumatism Association (ARA) classification criteria, who were recruited from primary and secondary care, attended a detailed study visit. The presence of palpable tophi was recorded. *SLC2A9 rs11942223, ABCG2 rs2231142* and *ABCG2 rs10011796* were genotyped. Data were analysed according to tophus status.

**Results:**

Compared to participants without tophi, those with tophi were older, had longer disease duration and higher serum creatinine, and were more likely to be of Māori or Pacific (Polynesian) ancestry. *SLC2A9 rs11942223* was not associated with tophi. However, the risk alleles for both *ABCG2* single nucleotide polymorphisms (SNPs) were present more frequently in those with tophi (OR (95% CI) 1.24 (1.02–1.51) for *rs2231142* and 1.33 (1.01–1.74) for *rs10011796*, *p* < 0.05 for both). The effect of *rs2231142* was limited to participants of Māori or Pacific ancestry (OR 1.50 (1.14–1.99), *p* = 0.004), with a significant effect observed in those of Western Polynesian ancestry only (OR 1.71 (1.07–2.72), *p* = 0.017). The *rs10011796* risk allele was strongly associated with tophi in the Western Polynesian group (OR 3.76 (1.61–8.77), *p* = 0.002), but not in the Eastern Polynesian group (OR 0.87 (0.52–1.46), *p* = 0.60) nor in the non-Polynesian group (OR 1.16 (0.81–1.66), *p* = 0.32). The *ABCG2* associations persisted in the Western Polynesian group after adjusting for serum urate, creatinine, and disease duration, and when including both *ABCG2* variants in the regression models.

**Conclusions:**

Variation in ABCG2 function may play a role in the development of tophaceous disease in some populations with high prevalence of severe gout.

**Electronic supplementary material:**

The online version of this article (doi:10.1186/s13075-017-1254-8) contains supplementary material, which is available to authorized users.

## Background

The gouty tophus consists of monosodium urate (MSU) crystal deposits surrounded by chronic granulomatous inflammatory tissue [1]. In people with gout, tophi contribute to musculoskeletal disability, joint damage and poor health-related quality of life, and are associated with increased risk of mortality [2–5]. Typically, tophi present as subcutaneous nodules many years after initial presentation with acute inflammatory flares [6]. Although advanced age, kidney disease and diuretic use have been reported to be risk factors for development of tophi (reviewed in [7]), few studies have systematically examined the features associated with tophaceous disease.

In the last decade, there has been major progress in understanding the genetic basis of hyperuricaemia and gout. Variants in two genes, *ABCG2* and *SLC2A9*, are consistently associated with hyperuricaemia and prevalent gout in many different populations [8–10].

Aotearoa New Zealand has a high prevalence of gout, with early onset, severe disease in Māori and Pacific people [11, 12]. The *SLC2A9 rs11942223* risk allele is strongly associated with prevalent gout in Māori and Pacific people living in Aotearoa New Zealand [13]. In contrast, the *ABCG2 rs2231142* risk allele (Q141K) has population-specific effects in Polynesian people, with a strong association with gout in people of Western Polynesian (Tonga, Samoa, Niue and Tokelau) ancestry, but a weak effect in people of Eastern Polynesian (New Zealand Māori and Cook Island Māori) ancestry [14].

Although the association with gout is well-established in many populations, it is unclear whether variants in these genes also contribute to phenotypic differences in people with gout. We have previously reported that a non-synonymous *SLC2A9* Arg265His variant is associated with tophi in New Zealand Māori with gout [15], and a Taiwanese study has reported an association between *ABCG2* Q141K and tophi in both Han Chinese and Taiwanese aboriginal people with gout [16]. The aim of this study was to examine the role of *SLC2A9* and *ABCG2* variants in tophaceous disease in people with gout.

## Methods

People with gout were recruited into the study, Genetics of Gout in Aotearoa, from primary and secondary care in Aotearoa New Zealand (n = 1778) [10]. The study was designed to identify genetic and clinical factors associated with gout. All participants in this analysis fulfilled the 1977 American Rheumatism Association (ARA) preliminary gout classification criteria [17]. The New Zealand Multi-Region Ethics Committee granted ethical approval (MEC/05/10/130) and all patients provided written informed consent.

All participants attended a study visit, which included a detailed clinical assessment. This included recording of age of onset of the first presentation with gout, flare frequency in the previous year (self-reported), medications, physical examination of weight and height, and serum creatinine testing. The presence of palpable tophi was assessed by experienced research assistants with training in the clinical assessment of gout. The highest recorded serum urate concentration was obtained from community electronic laboratory records. Presence of comorbid conditions (specifically cardiac disease, hypertension and type 2 diabetes mellitus) was recorded and verified using the electronic medical record. The estimated glomerular filtration rate (eGFR) was calculated using the modification of diet in renal disease formula [18].

*SLC2A9 rs11942223, ABCG2 rs2231142* and *ABCG2 rs10011796* single nucleotide polymorphisms (SNPs) were genotyped using TaqMan SNP genotyping assay technology (Applied Biosystems) [10]. *SLC2A9 rs11942223* and *ABCG2 rs2231142* were selected as variants consistently associated with gout in many populations, including Polynesian populations (reviewed in [9]). *ABCG2 rs10011796* was selected as an additional gout-associated *ABCG2* SNP [8], noting that this SNP has also been associated with allopurinol response in a USA study population [19]. The linkage disequilibrium (*r* ^2^) was <0.11 between the two *ABCG2* SNPs for all ancestral groups. Risk allele positivity was analysed for the *ABCG2* SNPs. However, owing to the high prevalence of the risk allele at *SLC2A9*, particularly in Polynesian people, minor allele (T) positivity was analysed instead.

Data were analysed in SPSS v22 (SPSS Inc., Chicago, IL, USA). Clinical and genetic features were analysed according to tophus status (tophi vs no tophi). Pearson’s chi-squared test (categorical variables) and the two-sample *t* test (continuous variables) were used to compare the two groups. *P* < 0.05 was considered significant for this analysis and was not corrected for multiple testing due to prior evidence of genetic effects in gout and tophus. For the detailed *ABCG2* analysis, logistic regression was performed on the presence/absence of the risk allele with tophus status, adjusted by potential confounders. Polynesian ancestry was separated into Western Polynesian (Tonga, Samoa, Niue or Tokelau) and Eastern Polynesian (Māori or Cook Island Māori) [14]. In the case of mixed Polynesian ancestry with Māori (n = 29), patients were analysed as Māori (Eastern Polynesian) as recommended by the New Zealand Ministry of Health.

## Results

### Clinical features

Of the 1778 participants with gout, there were 627 (35.3%) participants with at least one palpable tophus at the time of study examination. Compared to participants without tophi, those with tophi were older, had longer disease duration and higher serum creatinine, and were more likely to be of Māori or Pacific (Polynesian) ancestry (Table 1). Participants with tophi also reported more frequent gout flares and greater use of colchicine, prednisone, non-steroidal anti-inflammatory drugs and urate-lowering therapy.

**Table 1:** Clinical characteristics of participants

	All participants				Non-Polynesian ancestry				Māori or Pacific ancestry			
	With data, %	No tophi (n = 1151)	Tophi (n = 627)	*P*	With data, %	No tophi (n = 639)	Tophi (n = 288)	*P*	With data, %	No tophi (n = 512)	Tophi (n = 339)	*P*
Male sex, *n* (%)	100	955 (83.0%)	542 (86.4%)	0.06^a^	100	541 (84.7%)	245 (85.1%)	0.87^a^	100	414 (80.9%)	297 (87.6%)	0.01^a^
Age, years	99.5	57 (14)	58 (14)	0.52^b^	100	62 (14)	64 (13)	0.02^b^	100	52 (13)	53 (13)	0.50^b^
Duration of gout, years	98.3	13 (12)	17 (12)	<0.001^b^	98.7	14 (13)	16 (12)	<0.001^b^	97.8	11 (11)	18 (12)	<0.001^b^
Number of gout flares in previous year	95.9	4.8 (8.4)	7.9 (11.9)	<0.001^b^	95.4	3.8 (7.5)	6.2 (10.3)	<0.001^a^	96.5	6.0 (9.2)	9.4 (13.0)	<0.001^b^
Māori or Pacific ancestry, *n* (%)	100	512 (44.5%)	339 (54.1%)	<0.001^a^								
Body mass index, kg/m^2^	98.4	32.9 (8.05)	33.0 (7.66)	0.78^b^	98.5	30.31 (7.19)	29.86 (5.50)	0.35^b^	98.4	36.13 (7.90)	35.72 (8.20)	0.47^b^
Cardiac disease, *n* (%)	99.4	353 (30.9%)	221 (35.3%)	0.06^a^	99.7	203 (31.9%)	116 (40.3%)	0.01^a^	99.2	150 (29.6%)	105 (31.1%)	0.66^a^
Diabetes, *n* (%)	98.6	217 (19.2%)	138 (22.2%)	0.14^a^	99.4	92 (14.5%)	51 (17.7%)	0.22^a^	97.8	125 (25.1%)	87 (26.0%)	0.76^a^
Alcohol intake, servings per week	98.3	6.1 (10.8)	5.4 (9.3)	0.15^b^	99.4	6.9 (9.9)	6.6 (9.9)	0.62^b^	97.9	5.1 (11.8)	4.4 (8.5)	0.31^b^
Diuretic use, *n* (%)	100	285 (24.8%)	173 (27.6%)	0.06^a^	100	157 (24.6%)	85 (29.5%)	0.06^a^	100	128 (25.0%)	88 (26.0%)	0.57^a^
Colchicine use, *n* (%)	94.4	547 (51.2%)	406 (67.3%)	<0.001^a^	97.1	275 (44.6%)	168 (59.7%)	<0.001^a^	91.4	272 (53.1%)	238 (70.2%)	<0.001^a^
Prednisone use, *n* (%)	95.1	433 (40.1%)	322 (53.0%)	<0.001^a^	97.5	214 (34.5%)	141 (49.6%)	<0.001^a^	92.5	219 (47.6%)	181 (55.9%)	0.02^a^
NSAID use, *n* (%)	97.0	877 (78.6%)	499 (81.1%)	0.21^a^	98.2	470 (75.2%)	224 (78.8%)	0.23^a^	95.8	400 (83.2%)	275 (83.4%)	0.97^a^
Urate-lowering therapy, *n* (%)	99.5	829 (72.5%)	541 (86.4%)	<0.001^a^	99.5	441 (69.6%)	244 (84.7%)	<0.001^a^	99.5	388 (76.2%)	297 (87.9%)	<0.001^a^
Creatinine at time of recruitment, mmol/L	98.9	111 (57)	120 (57)	<0.001^b^	99.1	109 (45)	121 (57)	<0.001^b^	98.1	114 (68)	120 (57)	0.22^b^
eGFR, mL/min/1.73 m^2^	98.7	65 (20)	61 (22)	<0.001^b^	99.1	64 (20)	59 (22)	<0.001^b^	98.1	65 (21)	63 (21)	0.09^b^
Serum urate at time of recruitment, mmol/L	100	0.42 (0.11)	0.41 (0.12)	0.76^b^	100	0.40 (0.11)	0.40 (0.11)	0.59^b^	100	0.44 (0.11)	0.42 (0.13)	0.08^b^
Highest recorded serum urate, mmol/L	100	0.55 (1.11)	0.55 (0.13)	0.97^b^	100	0.49 (0.13)	0.52 (0.12)	<0.001^b^	100	0.62 (1.65)	0.57 (0.12)	0.59^b^

Unless specified, data are presented as mean (SD). ^a^ *P* value from Pearson’s chi-squared test. ^b^Two-sided *p* value from two-sample *t* test with equal variances. *NSAID* non-steroidal anti-inflammatory drugs, *eGFR* estimated glomerular filtration rate

In the 851 participants of Māori or Pacific ancestry, there were 339 participants (39.8%) with at least one palpable tophus at the time of the study examination. In this group, those with tophi were more likely to be male and had longer disease duration (Table 1). In the 927 participants who were not of Polynesian ancestry, there were 288 participants (31.1%) with at least one palpable tophus at the time of the study examination. In this group, those with tophi were older, and had longer disease duration, higher serum creatinine and higher ever-recorded serum urate concentration (Table 1).

### Genetic analysis

There was no difference in the frequency of the minor (protective) allele for *SLC2A9 rs11942223* between the group with tophi and the group without tophi for the entire sample set (*p* = 0.14) (Table 2 and Additional file 1). Similar findings were observed in the participants of Māori or Pacific ancestry and in the participants who were not of Polynesian ancestry (Table 2 and Additional file 1).

**Table 2:** *SLC2A9* and *ABCG2* genotype frequency in the entire group and in ancestral groups according to tophus status

	All participants				Non-Polynesian ancestry				Māori or Pacific ancestry			
	With data, %	No tophi (n = 1151)	Tophi (n = 627)	*P* ^a^	With data, %	No tophi (n = 639)	Tophi (n = 288)	*P* ^a^	With data, %	No tophi (n = 512)	Tophi (n = 339)	*P* ^a^
*SLC2A9 rs11942223* protective allele (C) present, *n* (%)	100	204 (17.7%)	94 (15.0%)	0.14	100	168 (26.3%)	69 (24.0%)	0.45	100	36 (7.0%)	25 (7.4%)	0.85
*ABCG2 rs2231142* risk allele (T) present, *n* (%)	100	483 (42.0%)	296 (47.2%)	0.033	100	263 (41.2%)	116 (40.3%)	0.80	100	220 (43.0%)	180 (53.1%)	0.004
*ABCG2 rs10011796* risk allele (T) present, *n* (%)	97.9	917 (81.7%)	529 (85.6%)	0.039	97.5	490 (78.9%)	230 (81.3%)	0.41	98.2	427 (85.2%)	299 (89.3%)	0.092

^a^ *P* value from Pearson’s chi-squared test

In contrast, in the entire group, the risk alleles for both *ABCG2* SNPs were present more frequently in those with tophi (odds ratio (OR) (95% CI) 1.24 (1.02–1.51) for *rs2231142* and 1.33 (1.01–1.74) for *rs10011796, p* < 0.05 for both (Table 2 and Additional file 1)). Analysis of *ABCG2* risk allele frequencies according to ancestry demonstrated that the effect of *rs2231142* was limited to participants of Māori or Pacific ancestry (OR 1.50 (1.14–1.99), *p* = 0.004) (Table 2 and Additional file 1), with a significant effect observed in those of Western Polynesian ancestry (OR 1.71 (1.07–2.72), *p* = 0.017) but not in Eastern Polynesian ancestry (OR 1.28 (0.84–1.96), *p* = 0.25) (Table 3 and Additional file 1). The *rs10011796* risk allele was also strongly associated with tophi in the Western Polynesian group (OR 3.76 (1.61–8.77), *p* = 0.002), but not in the Eastern Polynesian group (OR 0.87 (0.52–1.46), *p* = 0.60) nor in the non-Polynesian group (OR 1.16 (0.81–1.66), *p* = 0.32) (Table 3 and Additional file 1). The *ABCG2* associations persisted in the Western Polynesian group when including the other *ABCG2* variant in the regression model; the age and sex adjusted OR for *rs2231142* was 1.64 (1.101–2.65), *p* = 0.045, and for *rs10011796* it was 3.60 (1.53–8.44), *p* = 0.003.

**Table 3:** *SLC2A9* and *ABCG2* genotype frequency in Polynesian ancestry subsets according to tophus status

	Eastern Polynesian ancestry				Western Polynesian ancestry			
	With data, %	No tophi (n = 291)	Tophi (n = 173)	*p* ^a^	With data, %	No tophi (n = 221)	Tophi (n = 166)	*p* ^a^
*SLC2A9 rs11942223* protective allele (C) present, *n* (%)	100	24 (8.2%)	17 (9.8%)	0.56	100	12 (5.4%)	8 (4.8%)	0.79
*ABCG2 rs2231142* risk allele (T) present, *n* (%)	100	70 (24.1%)	50 (28.9%)	0.25	100	150 (67.9%)	130 (78.3%)	0.023
*ABCG2 rs10011796* risk allele (T) present, *n* (%)	98.3	242 (84.9%)	142 (83.0%)	0.60	98.2	185 (85.6%)	157 (95.7%)	0.001

^a^ *P* value from Pearson’s chi-squared test

The *ABCG2* associations also persisted in the Western Polynesian group after adjusting for age, sex, highest recorded serum urate, serum creatinine and disease duration; the multivariate OR for *rs2231142* was 1.66 (1.01–2.75), *p* = 0.048, and for *rs10011796* was 3.23 (1.35–7.76), *p* = 0.009 (Table 4). Similar results for both *ABCG2* SNPs were observed in participants of Māori or Pacific ancestry with at least three Polynesian grandparents, with generally higher ORs (Table 5).

**Table 4:** *ABCG2* analysis according to ancestry, with adjustment for potential confounders

*ABCG2* variant	Risk allele		All participants (n = 1778)		Non-Polynesian ancestry (n = 927)		Māori or Pacific ancestry (n = 851)		Eastern Polynesian ancestry (n = 464)		Western Polynesian ancestry (n = 387)	
			Allelic OR (95% CI)	*P*	Allelic OR (95% CI)	*P*	Allelic OR (95% CI)	*P*	Allelic OR (95% CI)	*P*	Allelic OR (95% CI)	*P*
*rs2231142*	T	Unadjusted	1.24 (1.02–1.51)	0.033	0.97 (0.73–1.28)	0.80	1.50 (1.14–1.99)	0.004	1.28 (0.84–1.96)	0.25	1.71 (1.07–2.72)	0.017
		Adjusted for age, sex, ancestry^a^	1.30 (1.06–1.58)	0.012	0.99 (0.74–1.31)	0.93	1.51 (1.14–2.01)	0.004	1.24 (0.81–1.91)	0.32	1.79 (1.12–2.87)	0.015
		Adjusted for age, sex, ancestry^a^, highest recorded urate, serum creatinine and disease duration	1.16 (0.94–1.43)	0.17	0.97 (0.72–1.30)	0.83	1.37 (1.01–1.85)	0.042	0.91 (0.57–1.45)	0.70	1.66 (1.01–2.75)	0.048
*rs10011796*	T	Unadjusted	1.33 (1.01–1.74)	0.040	1.16 (0.81–1.66)	0.41	1.44 (0.94–2.20)	0.093	0.87 (0.52–1.46)	0.60	3.76 (1.61–8.77)	0.002
		Adjusted for age, sex, ancestry^a^	1.31 (0.997–1.72)	0.052	1.18 (0.83–1.69)	0.36	1.45 (0.95–2.22)	0.089	0.87 (0.52–1.47)	0.61	3.75 (1.60–8.77)	0.002
		Adjusted for age, sex, ancestry^a^, highest recorded urate, serum creatinine and disease duration	1.25 (0.94–1.65)	0.13	1.12 (0.77–1.62)	0.55	1.39 (0.88–2.18)	0.16	0.82 (0.47–1.43)	0.49	3.23 (1.35–7.76)	0.009

^a^Adjusted by ancestry for analysis in “All participants”

**Table 5:** *ABCG2* analysis in Māori or Pacific participants who had at least three Polynesian grandparents, according to tophus status

*ABCG2* variant	Risk allele		Māori or Pacific ancestry (n = 689)		Eastern Polynesian ancestry (n = 323)		Western Polynesian ancestry (n = 366)	
			Allelic OR (95% CI)	*P*	Allelic OR (95% CI)	*P*	Allelic OR (95% CI)	*P*
*rs2231142*	T	Unadjusted	1.77 (1.30–2.42)	<0.001	1.65 (0.98–2.78)	0.059	1.77 (1.10–2.87)	0.020
		Adjusted for age and sex	1.76 (1.28–2.42)	<0.001	1.60 (0.95–2.69)	0.077	1.86 (1.14–3.04)	0.013
		Adjusted for age, sex, highest recorded urate, serum creatinine and disease duration	1.63 (1.17–2.28)	0.004	1.29 (0.74–2.26)	0.37	1.70 (1.01–2.86)	0.045
*rs10011796*	T	Unadjusted	1.73 (1.04–2.88)	0.036	0.90 (0.47–1.69)	0.73	4.61 (1.73–12.26)	0.002
		Adjusted for age and sex	1.74 (1.04–2.91)	0.034	0.91 (0.48–1.73)	0.78	4.58 (1.72–12.22)	0.002
		Adjusted for age, sex, highest recorded urate, serum creatinine and disease duration	1.72 (1.002–2.95)	0.049	0.89 (0.45–1.77)	0.75	3.90 (1.42–10.68)	0.008

## Discussion

This study provides evidence that variation in ABCG2 function may play a role in the development of tophaceous disease in some populations with high prevalence of severe gout. This effect appears to be independent of other risk factors such as serum urate concentrations or disease duration.

High prevalence of gout has been reported in indigenous Māori and in Pacific people residing in Aotearoa New Zealand, with contemporary rates of 11.7% in Māori men and 13.5% in Pacific men, compared to 3.7% in European men [11]. Our study has shown higher prevalence of tophaceous disease in people with gout of Māori or Pacific ancestry. Consistent with prior studies [6, 20, 21], we have also demonstrated that disease duration is associated with tophaceous disease in people with gout. Although serum urate concentrations were associated with tophaceous disease in participants of non-Polynesian ancestry, this association was not observed in the participants of Māori or Pacific ancestry. This study has also highlighted the impact of disease in patients with tophi, with more frequent flares and greater use of anti-inflammatory therapy, compared to people with gout without tophi.

The key finding of this study is the ancestry-specific differences in the association between *ABCG2* variants and tophaceous disease in people with gout. In particular, the associations with the *ABCG2* SNPs were observed consistently only in the Western Polynesian ancestral group. Association of a similar effect size between *rs2231142* (Q141K) and tophi has been reported in both Han and aboriginal Taiwanese people with gout (OR 1.51 and 1.50, respectively, for the 141 K allele) [16]. Other data also support the ancestry-specific effects of *ABCG2* in gout; we have previously reported that compared to people without gout, the *rs2231142* risk allele (141 K) is associated with susceptibility to gout in New Zealanders of European and Western Polynesian ancestry, but not in those of Eastern Polynesian ancestry [14]. Importantly, the frequency of the *rs2231142* risk allele is substantially higher in people of Western Polynesian ancestry, compared with the other ancestral groups: in a prior case-control study comparing people with gout with control participants with gout [14], the minor allele frequency in people unaffected by gout was 27.5% in Western Polynesians, 9.0% in Eastern Polynesians and 12.6% in Europeans; the minor allele frequency in people with gout was 51.9% in Western Polynesians, 10.7% in Eastern Polynesians and 24.2% in Europeans.

We also report a novel association between another *ABCG2* SNP *rs10011796* and tophi in people with gout who are of Western Polynesian ancestry. This ancestry-specific association between tophi and *rs10011796* was independent of *rs2231142* (Q141K). Together with *rs2231142*, this SNP has been associated with allopurinol response in a USA study population of people with gout [19]. Of note, the strong effect of *rs10011796* on allopurinol response in the USA study was attenuated after adjusting for ancestry, further suggesting ancestry-specific effects [19]. The ancestry-specific effects at *ABCG2* may relate to different haplotypic backgrounds between the Western Polynesian, European and Eastern Polynesian populations. This possibility could be evaluated by specific resequencing of this locus in people of Polynesian ancestry.

The mechanisms of association between *ABCG2* and tophaceous disease in people with gout are currently unclear. Both *SLC2A9* and *ABCG2* are associated with hyperuricaemia in the general population, and the lack of an observed association between tophaceous disease and *SLC2A9 rs11942223* suggests that the association between *ABCG2* and tophaceous disease in people with gout are not entirely due to effects on serum urate concentrations. This conclusion is further supported by the persistent associations with *ABCG2* after adjusting for serum urate and disease duration. The *ABCG2* risk alleles have been associated with allopurinol resistance, and it is possible that these variants lead to relative hyperuricaemia even with urate-lowering therapy, increasing the risk of tophaceous disease [19, 22]. ABCG2 (also known as breast cancer resistance protein (BCRP)) is ubiquitously expressed (reviewed in [23]), and it is also possible that altered ABCG2 function may regulate other factors contributing to tophus formation, such as promotion of crystal formation or the inflammatory response to deposited crystals.

## Conclusions

In our study, the association between *ABCG2* variants and tophaceous disease persisted in people with gout of Western Polynesian ancestry even after adjusting for potential confounders such as highest recorded urate, disease duration and serum creatinine. These findings raise the possibility that the relationship between *ABCG2* and tophaceous disease are independent of the effects of prolonged hyperuricaemia.

## Additional file

Additional file 1: Full *SLC2A9* and *ABCG2* genotype distributions according to tophus status. (PDF 42 kb)

## Abbreviations

ABCG2: ATP-binding cassette sub-family G member 2
ARA: American Rheumatism Association
eGFR: estimated glomerular filtration rate
MSU: Monosodium urate
OR: Odds ratio
SLC2A9: Solute carrier family 2, facilitated glucose transporter member 9
SNP: Single nucleotide polymorphism
