# Supplementary material for: Population-specific association between ABCG2 variants and tophaceous disease in people with gout
Source: Arthritis Res Ther. 2017 Mar 7;19:43. doi: 10.1186/s13075-017-1254-8 (PMC5341474; doi:10.1186/s13075-017-1254-8)
Supplement: Additional file 1: — Full SLC2A9 and ABCG2 genotype distributions according to tophus status. (PDF 42 kb) [file 13075_2017_1254_MOESM1_ESM.pdf]

Additional File 1. Full *SLC2A9* and *ABCG2* genotype distributions according to tophus status.

	Allele (A1/A2)	Genotype		
SLC2A9 rs11942223	**T/C**	**T/T**	**T/C**	**C/C**
All, no tophi (n=1151)		947 (82.2%)	189 (16.4%)	15 (1.3%)
All, tophi (n=627)		533 (85.0%)	89 (14.2%)	5 (0. 8%)
Non-Polynesian ancestry, no tophi (n=639)		471 (73.7%)	155 (24.3%)	13 (2.0%)
Non-Polynesian ancestry, tophi (n=288)		219 (76.0%)	66 (22.9%)	3 (1.0%)
Māori or Pacific ancestry, no tophi (n=512)		476 (93.0%)	34 (6.6%)	2 (0.4%)
Māori or Pacific ancestry, tophi (n=339)		314 (92.6%)	23 (6.8%)	2 (0.6%)
Eastern Polynesian ancestry, no tophi (n=291)		267 (91.8%)	23 (7.0%)	1 (0.3%)
Eastern Polynesian ancestry, tophi (n=173)		156 (90.2%)	15 (8.7%)	2 (1.2%)
Western Polynesian ancestry, no tophi (n=221)		209 (94.6%)	11 (5.0%)	1 (0.5%)
Western Polynesian ancestry, tophi (n=166)		158 (95.2%)	8 (4.8%)	0 (0%)
ABCG2 rs2231142	**G/T**	**G/G**	**G/T**	**T/T**
All, no tophi (n=1151)		668 (58.0%)	393 (34.1%)	90 (7.8%)
All, tophi (n=627)		331 (52.8%)	228 (36.4%)	68 (10.8%)
Non-Polynesian ancestry, no tophi (n=639)		376 (59.3%)	222 (34.7%)	41 (6.4%)
Non-Polynesian ancestry, tophi (n=288)		172 (75.4%)	94 (32.6%)	22 (7.6%)
Māori or Pacific ancestry, no tophi (n=512)		292 (57.0%)	171 (33.4%)	49 (9.5%)
Māori or Pacific ancestry, tophi (n=339)		159 (46.9%)	134 (39.5%)	46 (13.6%)
Eastern Polynesian ancestry, no tophi (n=291)		221 (75.9%)	68 (23.4%)	2 (0.7%)
Eastern Polynesian ancestry, tophi (n=173)		123 (71.1%)	45 (26.0%)	5 (2.9%)
Western Polynesian ancestry, no tophi (n=221)		71 (32.1%)	103 (46.6%)	47 (21.2%)
Western Polynesian ancestry, tophi (n=166)		36 (21.7%)	89 (53.6%)	41 (24.7%)
ABCG2 rs10011796	**T/C**	**TT**	**CT**	**CC**
All, no tophi (n=1122)		408 (36.4%)	509 (45.3%)	205 (18.3%)
All, tophi (n=618)		236 (38.2%)	293 (47.4%)	89 (14.4%)
Non-Polynesian ancestry, no tophi (n=621)		205 (33.0%)	285 (45.9%)	131 (21.1%)
Non-Polynesian ancestry, tophi (n=283)		101 (35.7%)	129 (45.6%)	53 (18.7%)
Māori or Pacific ancestry, no tophi (n=501)		203 (40.5%)	224 (44.7%)	74 (14.8%)
Māori or Pacific ancestry, tophi (n=335)		135 (40.3%)	164 (49.0%)	36 (10.7%)
Eastern Polynesian ancestry, no tophi (n=285)		107 (37.5%)	135 (47.4%)	43 (15.1%)
Eastern Polynesian ancestry, tophi (n=171)		55 (32.2%)	87 (50.9%)	29 (17.0%)
Western Polynesian ancestry, no tophi (n=216)		96 (44.4%)	89 (41.2%)	31 (14.4%)
Western Polynesian ancestry, tophi (n=164)		80 (48.8%)	77 (47.0%)	7 (4.2%)
